# Petunidin-Based Anthocyanin Relieves Oxygen Stress in *Lactobacillus plantarum* ST-III

**DOI:** 10.3389/fmicb.2020.01211

**Published:** 2020-07-07

**Authors:** Minghui Yan, Bing-hua Wang, Xiaofei Fu, Min Gui, Guojiao Wang, Lei Zhao, Ruiying Li, Chunping You, Zhenmin Liu

**Affiliations:** ^1^ State Key Laboratory of Dairy Biotechnology, Shanghai Engineering Research Center of Dairy Biotechnology, Dairy Research Institute, Bright Dairy & Food Co., Ltd., Shanghai, China; ^2^ Department of Clinical Laboratory, Central Laboratory, Jing’an District Center Hospital of Shanghai, Fudan University, Shanghai, China; ^3^ Bright Farming Co., Ltd., Shanghai, China; ^4^ School of Life Sciences, Shanghai University, Shanghai, China; ^5^ College of Food Science and Technology, Shanghai Ocean University, Shanghai, China

**Keywords:** *Lactobacillus plantarum*, petunidin, anthocyanin, *Lycium ruthenicum* Murray, oxygen stress, probiotics, milk

## Abstract

Application of probiotics in the food industry has been hampered by their sensitivity to challenging conditions that reduce their vitality in food matrices. A lot of attempts have been made to promote the growth of these probiotics in the aspect of nutrition demands. Among the other adverse conditions, oxygen stress can restrict the growth of probiotics and has not yet been paid enough attention to. In this study, the effect of a petunidin-based anthocyanin (ACN) on the growth of probiotic *Lactobacillus plantarum* ST-III was investigated under oxygen stress. The growth of ST-III was analyzed through spot assay on agar plates as well as plating-based enumeration of the viable cells in the liquid culture. Results indicated that ACN could efficiently improve the growth of ST-III under oxygen stress, whereas no effect was observed in the absence of oxygen stress. Further investigations indicated that ACN reduced the oxido-reduction potential of the culture; meanwhile, it exerted a positive transcriptional regulation on the thioredoxin system of ST-III, leading to a decrease in reactive oxygen species accumulation within the cells. Moreover, ACN enabled the growth of ST-III in reconstituted skim milk and promoted the formation of milk clots. These results revealed the role of a petunidin-based ACN in oxygen stress relief and highlighted its potential in manufacture and preservation of *L. plantarum*-based dairy products.

## Introduction

According to the expert consensus document of the International Scientific Association for Probiotics and Prebiotics (ISAPP), probiotics are currently defined as live microorganisms that, when administered in adequate amounts, confer a health benefit on the host (Hill et al., 2014). To date, randomized controlled trials have shown health benefits of probiotics across a range of populations, from healthy individuals to those with acute and chronic diseases (Huang et al., 2015; Papadimitriou et al., 2015; Mu et al., 2018). Based on these studies, probiotic products have been developed in the form of drugs, foods, or drinks such as yoghurts and dietary supplements. *Lactobacillus* and *Bifidobacterium* are the two main genera on the list of probiotics developed so far. Unfortunately, many of the most studied probiotic strains are oxygen-sensitive (Mills et al., 2011; Hill et al., 2014; Bhattarai et al., 2019), and the toxicity of oxygen has become a main factor that restricts the application of these oxygen-sensitive probiotics (Ruiz et al., 2012; Maresca et al., 2018). *Lactobacillus plantarum* is a well-documented probiotic species and several strains have shown health benefits such as cholesterol-lowering, anti-inflammatory effect, gut microbiota modulation, and protection against heavy metals (Zhai et al., 2016; Wu et al., 2017; Dan et al., 2018; Malik et al., 2018). Despite its probiotic potentials, *L. plantarum* lacks the enzymes to deal with toxic oxygen-derived compounds and therefore is sensitive to oxygen stress (Papadimitriou et al., 2016). Several studies have demonstrated that some strains are able to grow under aerobic (AE) conditions in complex and synthetic media (Pedersen et al., 2012; Guidone et al., 2013; Zotta et al., 2017); however, *L. plantarum* strains are generally hard to grow under milk conditions, probably due to their weak proteolytic ability (Ma et al., 2016; Behera et al., 2018). *L. plantarum* ST-III is a probiotic strain with a lot of beneficial effects, including cholesterol-lowering and gut microbiota modulation (Liu et al., 2008; Yan et al., 2018a). Like its counterparts, ST-III is sensitive to oxygen and fails to grow in milk under air conditions, restricting its applications in the dairy industry (Siragusa et al., 2014; Jiang et al., 2016).

Anthocyanin (ACN) is one of the subclasses of phenolic phytochemicals characterized by its antioxidant properties. Naturally occurring ACNs are mostly in the form of anthocyanidin glycosides. Chemically, anthocyanidins are grouped into 3-hydroxyanthocyanidins, 3-deoxyanthocyanidins, and *O*-methylated anthocyanidins; the most common types of anthocyanidins are cyanidin, delphinidin, pelargonidin, peonidin, petunidin, and malvidin (Khoo et al., 2017). ACNs are potential pharmaceutical ingredients that give various beneficial health effects, as they possess anti-oxidative and antimicrobial activities, improve visual and neurological health, and protect against cardiovascular disease (Wallace and Giusti, 2015; Khoo et al., 2017). It is suggested that these health benefits could be attributed to the potent antioxidant properties of ACNs (Wallace and Giusti, 2015; Khoo et al., 2017). Recent studies reported the use of ACNs of different origins to improve the growth of probiotic strains, including *Bifidobacterium* and *Lactobacillus* (Sun et al., 2018; Zhu et al., 2018). However, these studies were carried out under anaerobic (AN) conditions and the effect of oxygen was not been taken into consideration. Besides, previous studies are mostly performed with sequencing-based methods, whereas culture-based analysis is required for full clarification of the interactions between ACNs and microbes. Culture-based studies on this topic will not only provide mechanistic insights into the modulation of intestinal microbiota by ACNs, but also enable solutions for growth and viability of probiotics in food stress.

This study was designed to investigate the effect of a petunidin-based ACN from *Lycium ruthenicum* Murray on the growth of *L. plantarum* ST-III under oxygen stress. As far as we are acknowledged, this is the first report on the interaction between ACNs and probiotics. Moreover, oxygen stress relief by ACN enabled the growth of ST-III in milk, indicating a potential of ACN in manufacture of *L. plantarum*-based dairy products.

## Materials and Methods

### Preparation of ACN From *Lycium ruthenicum* Murray

The dried fruits of *L. ruthenicum* (Black goji) were obtained from a local market in Xi’ning, Qinghai, China. The procedures for preparation of ACN were performed according to the reported method (Yan et al., 2018c) with some modifications. In brief, 50.0 g of dried fruits of *L. ruthenicum* were extracted with 80% aqueous ethanol (vol/vol, with 0.1% formic acid) at a ratio of 1:40 (wt/vol) in 50°C water bath for 3 h. The extract was then filtered through a 0.45 μm cellulose filter and concentrated with vacuum rotary evaporator. The concentrate was then diluted and loaded onto a column of wet-packed AB-8 resin. The column was washed with two times bed volume of deionized water and then eluted with 85% aqueous ethanol solution (vol/vol, pH 2.0). The effluent was collected, concentrated, and freeze-dried, affording the ACN for the following experiments.

### Analysis of the ACN

The ACN was analyzed by High Performance Liquid Chromatography (HPLC) coupled to mass spectrometer and the ACN content was determined by spectrophotometric pH differential method as described previously (Tang et al., 2017). Briefly, 0.4 ml of sample was individually mixed with 3.6 ml and 0.025 M potassium chloride (pH 1.0) and 0.4 M sodium acetate solution (pH 4.5), and the absorbance of the mixed samples at pH 1.0 and 4.5 were measured at 519 nm against distilled water as the blank by using a spectrophotometer. The ACNs content was calculated according to the previous description (Tang et al., 2017) and expressed as cyanindin-3-glucoside equivalence.

### *L. plantarum* Strains

*L. plantarum* ST-III (CGMCC 0847) was provided by the State Key Laboratory of Dairy Biotechnology, Bright Dairy & Food Co., Ltd., China. The bacteria strain was routinely activated in MRS medium (Merck, Germany) at 30°C in a Whitley A35 Anaerobic Workstation (Don Whitley Scientific, United Kingdom) filled with a mixture gas of N_2_:H_2_:CO_2_ = 85:10:5 (vol/vol). The activated culture was then subjected to growth analysis under oxygen stress.

### Spot Assays

Spot assays were carried out on TYC plates (15.0 g/L tryptone, 5.0 g/L yeast extract, 0.2 g/L L-cystine, 0.1 g/L sodium sulphate, 1.0 g/L sodium chloride, 0.8 g/L disodium phosphate anhydrous, 2.0 g/L sodium bicarbonate, 12.0 g/L sodium acetate anhydrous, 50.0 g/L sucrose, and 15 g/L agar, pH 7.4) as described previously in AE and AN conditions (Bueno et al., 2018; Yan et al., 2018b). In brief, activated culture of *L. plantarum* ST-III was 1:50 diluted into fresh MRS medium and cultivated anaerobically to an OD595 of ∼0.8. Cells were harvested from 10 ml culture by centrifugation at 5,000 rpm for 2 min. After two times wash, the cell pellets were resuspended and 10-fold serial dilutions of cell suspensions were prepared. Five microliter aliquots from each dilution were spotted onto TYC plates and incubated aerobically at 30°C for 3 days. Equivalent aliquots were spotted on MRS plates and incubated anaerobically at 30°C as control. Photos of agar plates were taken using a Molecular Imager Gel Doc XR system (Bio-Rad) equipped with a white-light trans-illuminator. The experiments were performed in triplicate (three independent biological replicates), and the representative results are shown.

### Liquid Culture Under Oxygen Stress

The growth of *L. plantarum* under oxygen stress was performed in liquid TYC medium under AE and AN conditions, as previously described (Yan et al., 2018b). Cell pellets from overnight culture were washed three times and resuspended into 50 ml fresh TYC medium (pH 7.4) in 250 ml shaken flasks to an OD_595_ of ∼0.2. The cultures were then incubated at 30°C under air conditions with agitation at 120 rpm for 24 h, and samples were taken at 0, 2, 4, 8, 12, and 24 h for enumeration of viable cells in the culture. To test its effect on the growth of *L. plantarum*, ACN was supplemented to a final concentration of 0.025% (wt/vol). Equivalent cultures were incubated anaerobically and the growth was compared with that under air conditions. The experiments were performed in triplicate, and the average values are shown with standard deviations.

### Enumeration of *L. plantarum*

The number of viable cells in the culture was determined through gradient dilutions and plating-based methodology, as described previously (Ashraf and Shah, 2011), with slight modifications. In brief, samples were taken at the indicated time points and serially diluted using sterile saline (9.0 g/L of NaCl) and plated on MRS plates. After 3 days cultivation in Whitley A35 Anaerobic Workstation at 35°C, plates with 30–300 colonies were counted and the numbers of viable cells were calculated and expressed as CFU/ml culture. The enumeration was carried out in triplicate and the average results are shown.

### ROS Determination

*L. plantarum* ST-III was cultivated in TYC medium under AE or AN conditions, as described above. After 12 h cultivation, bacteria cells were harvested by centrifugation at 5,000 rpm for 2 min and the level of reactive oxygen species (ROS) was determined using 2,7-dichlorodihydrofluorescein diacetate (H_2_DCF-DA; Beyotime Institute of Biotechnology, Haimen, China) according to the instructions provided by the manufacturer. In brief, around 1 × 10^6^ cells were collected and washed with phosphate-buffered solution (0.01 M phosphate, pH 7.4, PBS) and then treated with 10 mM H_2_DCF-DA dissolved in PBS at 37°C anaerobically for 20 min. After removal of H_2_DCF-DA and three times wash with PBS, the fluorescence intensity was monitored with excitation wavelength at 488 nm and emission wavelength at 525 nm on SpectraMax M5, Molecular Devices (San Jose, CA, United States).

For each sample, an equal amount of cells were sonicated and subjected to quantification of the total protein using the Bradford method. The fluorescence intensity was normalized with the total protein content, and the relative amount of ROS is expressed as DCF fluorescence intensity per milligram total protein, as described previously (Yan et al., 2018b). The experiments were performed in triplicate and the average values, as well as the standard deviations, are shown.

### Determination of Extracellular Oxido-Reduction Potential

*L. plantarum* ST-III was cultivated in TYC medium under air conditions and the extracellular oxido-reduction potential (ORP) was determined as described in previous studies (Zhu et al., 2014; Huang et al., 2017). In brief, after being cultured, 20 ml culture broth was filtered under vacuum through a 0.8-μm mixed cellulose ester membrane, and the filtrate was diluted appropriately to determine the ORP. ORP of the culture was detected at 12 and 24 h *via* an oxidation-reduction electrode (Leici, Shanghai), and the values were calibrated according to the reference electrode value (210 mV at 37°C).

### RNA Extraction and Transcription Analysis (qRT-PCR)

Bacterial cells were harvested by centrifugation at 8,000 × *g* for 10 min at 4°C and total RNA was extracted using RNeasy Mini kits (Qiagen). *cDNA* synthesis was performed using the FastQuant RT kit with gDNase (Tiangen) according to the manufacturer’s instructions. Quantitative real time-PCR (qRT-PCR) was performed with Taq SYBRGreen qPCR Premix on a 7500 Fast System (Applied Biosystems) according to the manufacturer’s instructions. The thermo-cycling conditions were 95°C for 10 min, followed by 40 cycles of 95°C for 15 s, 55°C for 30 s, and 60°C for 60 s according to a previous study (Zotta et al., 2012). The sequences of the primers are listed in Table 1. Three independent replicates of each sample were tested and the 2^−ΔΔCt^ method was used to calculate the expression levels of the target genes. 16S rRNA transcript was used as the internal standard for transcription analysis (Chen et al., 2019).

**Table 1:** Primers used in quantitative real time-PCR.

Name	Nucleotide sequence
16S rRNA	5'-CGCAAGGCTGAAACTCAAAGG-3' 5'-CTGACGACAACCATGCACCAC-3'
*trxB1*	5'-ATGGCAAAGAGTTACGACG-3' 5'-TTCAGAACCAGTCCCAATGAC-3'
*trxA2*	5'-ATGGTCGCAGCAACTACTG-3' 5'-TTATAGATATTGAGCTAAAGTTTG-3'
*ccpA*	5'-ATGGAAAAACAAACAGTA-3' 5'-GCATCATCGGAGTTCGTTAAAATG-3'

### The Growth of *L. plantarum* in Reconstituted Skim Milk

The reconstituted skim milk (RSM) was prepared, as previously described (Li et al., 2016). Skim milk powder (33.4% protein, 0.8% fat, 54.1% lactose, and 7.9% minerals, Fonterra Ltd., Auckland, New Zealand) was reconstituted in distilled water. The RSM was stirred with a Stirrer RW20 (IKA, Staufen, Germany) at 600 rpm for 5 min, and then heat-treated at 95°C for 15 min immediately. After cooling to room temperature (25°C), ACN was supplemented to a final concentration of 0.025% (wt/vol). The mixtures were then inoculated with *L. plantarum* ST-III (5 × 10^7^ CFU/ml) and incubated aerobically in a 30°C incubator. Samples were taken at the indicated time points and the viable cells were quantified with a plating-based methodology as described above. During the incubation, the formation of milk clots was investigated. Photos were taken at 48 h and the representative images of milk clots are shown.

For spot assays on milk plates, RSM agar plates (containing 1% skimmed milk powder and 1.5% agar, wt/vol) were exploited. Cell suspensions of ST-III were 10-fold serially diluted and spotted onto RSM agar plates with or without ACN. Growth was performed under air conditions at 30°C and photographs of the plates were taken after 72 h cultivation. All the experiments were performed in triplicate (three independent biological replicates).

## Results

### A Petunidin-Based Anthocyanin From *Lycium ruthenicum* Murray

The ACN was extracted from dried fruits of *L. ruthenicum* and purified, as described previously (Zheng et al., 2011) and the ACN was analyzed by HPLC coupled to mass spectrometer. Determination of the ACN content by pH differential method showed that the ACN obtained contains 3.7 g/L ACN, among which nearly 98% is ACN, as revealed by HPLC analysis. This result is consistent with previous studies (Zheng et al., 2011; Yan et al., 2018c).

### ACN Stimulated the Growth of *Lactobacillus plantarum* ST-III Under Oxygen Stress

**Figure 1:**
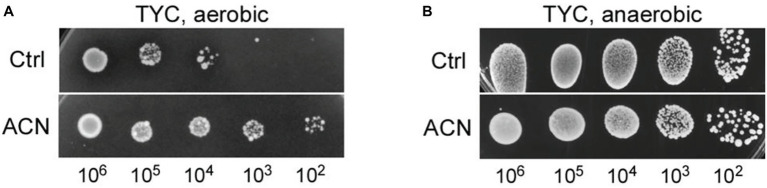
The growth of *Lactobacillus plantarum* ST-III under oxygen stress as reflected by spot assays on TYC-agar plates. **(A)** Cells of ST-III were suspended and serially diluted, the cell suspensions were then spotted onto TYC plates with or without anthocyanin (ACN) and cultivated aerobically at 30°C for 48 h. **(B)** For anaerobic (AN) control, equivalent plates were cultivated anaerobically. All the photographs of the agar plates were taken with a Molecular Imager Gel Doc XR system (Bio-Rad) equipped with a white-light trans-illuminator. The experiments were performed in triplicate, and the representative images are shown.

The effect of the ACN on the growth of ST-III was investigated under oxygen stress, a condition usually encountered in the food industry. Oxygen stress was simulated as previously described (Yan et al., 2018b) and the growth of ST-III was analyzed by spot assays. As shown in Figure 1A, the growth of ST-III was much more vigorous when ACN was supplemented (compare ACN with Ctrl). The ACN could promote the growth of ST-III by about two orders of magnitude, whereas no obvious effect was observed in the AN control (Figure 1B), suggesting that the ACN could efficiently promote the growth of ST-III under oxygen stress.

### Effect on the Growth of *L. plantarum* ST-III in Liquid Culture

**Figure 2:**
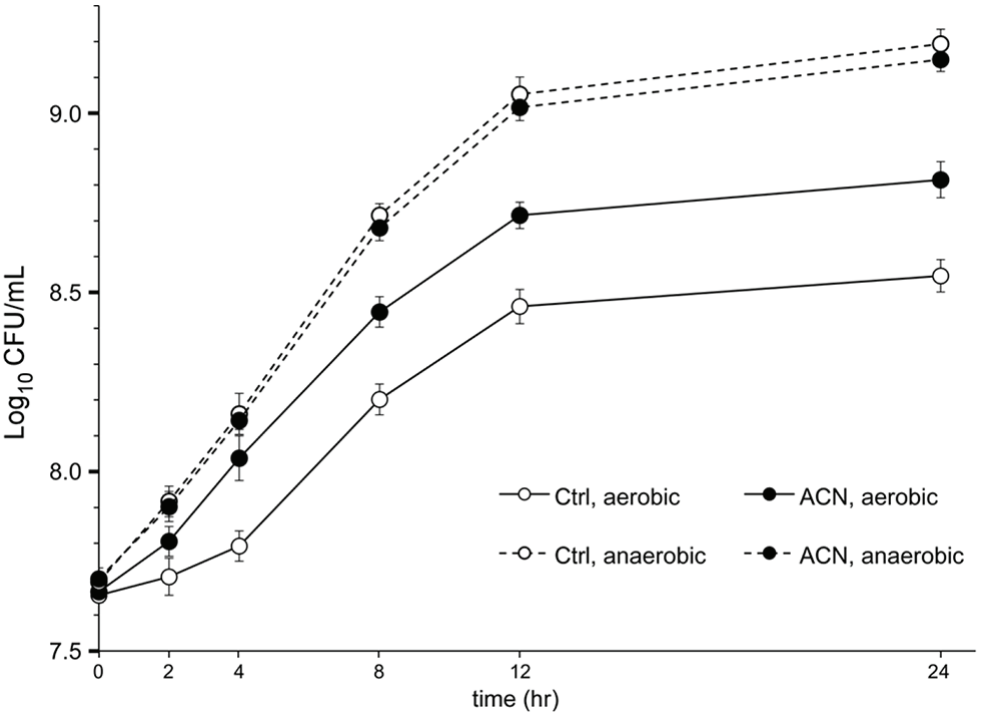
The growth of *L. plantarum* ST-III in liquid culture. *L. plantarum* ST-III was cultivated in liquid TYC medium with or without 0.025% ACN under aerobic (AE) and AN conditions (shown in solid and dashed lines, respectively). The growth of ST-III was estimated by the number of viable cells (CFU/ml) determined by plating-based method. Experiments were performed in triplicate and the average values, as well as the standard deviations, are included in the graphs.

The effect on the growth of *L. plantarum* ST-III was also examined in liquid TYC medium, as previously described (Yan et al., 2018b), with or without ACN. Bacterial growth was reflected by colony-forming units, and the growth of ST-III with 0.025% ACN was compared to that without ACN. As shown in Figure 2, cells of ST-III in liquid culture with ACN proliferated after a 2-h stagnation, whereas those without ACN experienced a growth stagnation of about 4 h. This trend was consistent with the oxygen stress level reported in a previous study (Yan et al., 2016). After 24 h cultivation, the growth advantage in ACN was still obvious. However, no such growth advantage was observed in the AN culture (Figure 2, dashed lines). These results indicated that ACN could effectively promote the growth of *L. plantarum* ST-III in liquid culture under oxygen stress. Note should be taken that the growth advantage endowed by ACN was not as obvious as that observed on agar plates. This might be explained by the lower intensity of oxygen stress in liquid culture, as revealed in previous studies (Raso et al., 2012; Laureys et al., 2018).

### Relief of the Oxygen Stress by ACN

**Figure 3:**
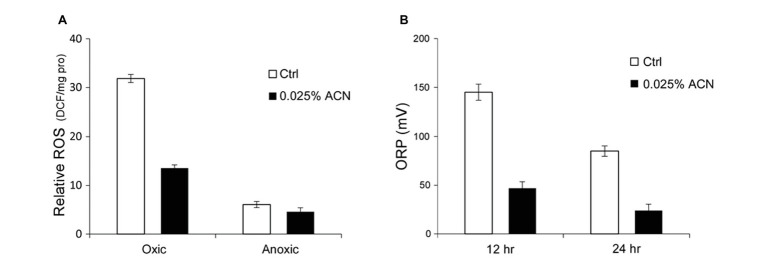
Reactive oxygen species (ROS) accumulation within cells of ST-III and oxido-reduction potential (ORP) of the culture. **(A)** Determination of ROS accumulation within the cells. *L. plantarum* ST-III was incubated anaerobically or in the presence of environmental oxygen. Samples with or without ACN were taken after 48 h incubation. The amount of ROS within the cells was determined using DCFH-DA as a probe and the results were normalized to total protein content (relative ROS expressed as DCF/mg pro). **(B)** ORP of the medium during cultivation. Culture of ST-III was sampled at the indicated time points and ORP was determined. The experiments were performed in triplicate and the average results, as well as the standard deviations, are included in the graphs.

The results above indicated that the growth improvement might be attained through relief of the oxygen stress. The extent of oxygen stress within ST-III cells inoculated with or without ACN was analyzed. Previous studies showed that a considerable amount of ROS accumulated within cells of lactic acid bacteria encountered with oxygen stress (Guidone et al., 2013; Imlay, 2015). Therefore, ROS accumulation within cells of ST-III was quantified to evaluate the intensity of oxygen stress. As shown in Figure 3A, after 12 h cultivation under oxygen stress, ST-III cells accumulated ROS at ~30 DCF/mg pro, whereas supplementation of 0.025% ACN could reduce the amount of ROS by more than half. This result was, in accordance with the growth advantage of ST-III with ACN, suggesting that the growth improvement by ACN might have been mediated by relief of the oxygen stress.

Additionally, the ORP of the culture, an essential physiological parameter for micro-aerobic fermentations (Liu et al., 2013), was determined. The results indicated that the ORP of the medium was obviously reduced by ACN (Figure 3B), consistent with the trend of ROS accumulation (Figure 3A). These results provided further evidence that the growth improvement by ACN was attained through relief of the oxygen stress.

### The Involvement of Thioredoxin (TRX) System

**Figure 4:**
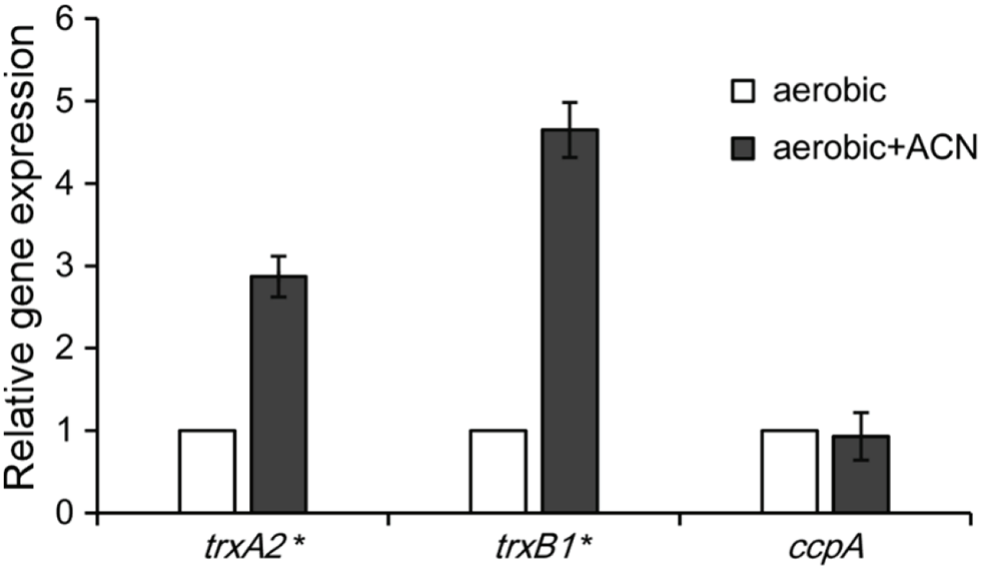
Quantitative real time-PCR (qRT-PCR) analysis of the oxidative stress-related genes. *L. plantarum* ST-III was cultivated under oxygen stress with and without ACN and the relative expression level of *trxA2*, *trxB1*, and *ccpA* was measured by qRT-PCR. The transcription level of each gene with ACN was expressed as fold to that without ACN. The results are shown as average value of three biological and technical replicates. Statistical significance is identified as ^*^ *p* < 0.05.

When encountered with oxidative stress, *L. plantarum* enhanced the expression of antioxidant enzymes such as those of the thioredoxin (TRX) system (Serrano et al., 2007). Previous studies identified *trxB1* and *trxA2* as two genes highly conserved within the *L. plantarum* strains that encode the main enzymes in the TRX system (Serrano et al., 2007; Zhai et al., 2020). qRT-PCR assays were performed to investigate the effect of ACN on the transcription of *trxB1* and *trxA2* in ST-III. As shown in Figure 4, both *trxA2* and *trxB1* were significantly up-regulated by ACN (by 2.87- and 4.65-fold, respectively), suggesting that the oxygen stress relief by ACN might be associated with the enhancement of TRX system. In addition, the transcription of *ccpA*, an important gene involved in the AE growth of *L. plantarum* (Zotta et al., 2012), was not affected.

### Oxygen Stress Relief by ACN Enabled the Aerobic Growth of ST-III on Milk Plates

**Figure 5:**
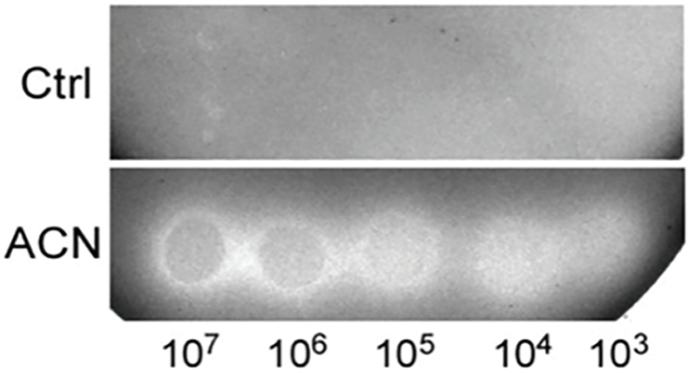
The growth of *L. plantarum* ST-III on RSM-agar plates. Cells of ST-III were suspended and serially diluted to the concentrations as indicated and spotted onto RSM-agar plates with or without 0.025% ACN. The plates were then incubated aerobically at 30°C. Photos were taken after 72 h incubation and the representative photos are shown.

Although they are widely seen in a lot of fermented foods, food conditions are generally regarded as stress conditions for *L. plantarum* and other lactic acid bacteria (Papadimitriou et al., 2016). The effect of ACN in relief of the oxygen stress led to the hypothesis that it might improve the growth of ST-III in food stress. Therefore, the effect of ACN on the growth of ST-III was evaluated by spot assays on RSM-agar plates under air conditions. As shown in Figure 5, whereas ST-III failed to grow on RSM-agar plate (as shown in Ctrl group), the supplementation of 0.025% ACN could efficiently stimulate the growth of ST-III (shown as bacteria spots in ACN group). This result suggested that relief of the oxygen stress by ACN might bypass the nutritional demand of ST-III and enable its growth on milk plates.

### ACN Improved the Growth of ST-III in Liquid RSM

**Figure 6:**
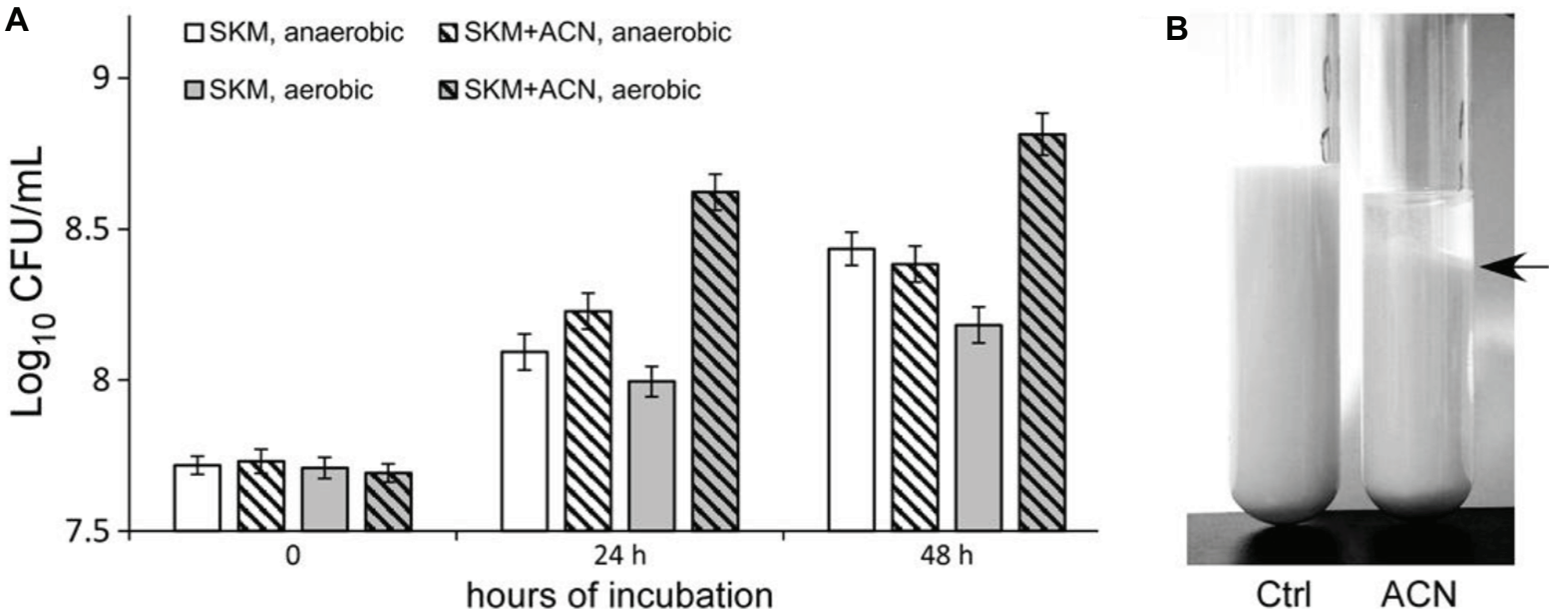
The growth of *L. plantarum* ST-III in liquid reconstituted skim milk (RSM). **(A)** *L. plantarum* ST-III was cultivated in liquid RSM under AE and AN conditions. The growth of ST-III was estimated by CFU/ml. Experiments were performed in triplicate, and the average values, as well as the standard deviations were included in the graphs. A slight inhibition of growth was observed; this might be attributed to the weak antimicrobial activity of ACN, as previous studies have shown the anti-microbial activity of polyphenol compounds (Daglia, 2012; Bouarab-Chibane et al., 2019). **(B)** Representative image of ST-III culture in RSM after 48 h AE incubation. Note that milk clot formation in ST-III culture with ACN was obvious (indicated by a black arrow on the right).

Subsequently, the effect of ACN on the growth of ST-III was investigated in liquid RSM. The viable cells in the liquid RSM culture with or without ACN were enumerated. As shown in Figure 6A, when 0.025% ACN was supplemented, ST-III underwent a much more vigorous growth in liquid RSM, as compared to that without ACN. After 48 h cultivation, ST-III could proliferate by more than 10-fold with ACN, whereas only 3-fold of proliferation was observed in the absence of ACN. However, no obvious growth improvement was observed under AN conditions (Figure 6A). Notably, after 48 h cultivation, milk clots formation by ST-III was evident in liquid RSM supplemented with ACN, whereas the group without ACN did not coagulate (Figure 6B). This is consistent with the proliferation of ST-III with ACN observed in Figure 6A. These results revealed that ACN is also effective in growth improvement of ST-III in liquid RSM, suggesting its potential in manufacture of *L. plantarum*-based dairy products.

## Discussion

Probiotics are defined as live microorganisms that, when administered in adequate amounts, confer a health benefit on the host (Hill et al., 2014). In order to exert a beneficial effect, a probiotic needs to be administered in sufficiently high numbers, and thus it is imperative that the probiotic survives cultivation, processing, and storage. *Lactobacillus* and *Bifidobacterium* are two important genera on the list of probiotics with well-established health benefits to human body (Hill et al., 2014; Fiocco et al., 2019). However, strains of both genera are generally sensitive to oxygen, and oxygen stress has become one of the most important abiotic factors that negatively affect their growth and survival (Papadimitriou et al., 2016; Fiocco et al., 2019). Oxygen, however, is prevalent in the process of manufacture and storage of probiotic products, making it an enormous threat to the performance of probiotics. Therefore, it is an urgent demand to develop techniques to protect these probiotic bacteria from oxygen toxicity.

*L. plantarum* is a well-documented probiotic species, and several strains have shown health benefits such as cholesterol-lowering, anti-inflammatory effect, gut microbiota modulation, and protection against heavy metals (Zhai et al., 2016; Wu et al., 2017; Dan et al., 2018). *L. plantarum* is a lactic acid bacterium found in diverse ecological niches, highlighting its particular capabilities of adaptation (Seddik et al., 2017). Therefore, it is employed as a model organism for academic research of lactic acid bacteria (Siezen and van Hylckama Vlieg, 2011; Seddik et al., 2017; Parlindungan et al., 2019), also in the area of stress response and tolerance (Fiocco et al., 2010; Papadimitriou et al., 2016; Arena et al., 2019; Zhao et al., 2019). Studies on *L. plantarum* have deepened the understanding of oxygen-tolerance in *Lactobacillus* and other probiotics (Archibald and Fridovich, 1981; Serrano et al., 2007; Papadimitriou et al., 2016).

The growth of probiotic *L. plantarum* in milk has become a critical issue in the dairy industry that hampers their extensive applications. According to previous studies, the growth of these probiotics in milk is restricted by factors including nutrients inadequacy, acid, and oxygen stress (Li et al., 2016; Fiocco et al., 2019). Moreover, this problem is further complicated by the interactions among these growth-restricting factors (Papadimitriou et al., 2016; Zhang et al., 2018).

ACN is one of the subclasses of phenolic phytochemicals widely spread in flowers, fruits, and vegetables with a wide variety of biological activities (Wallace and Giusti, 2015). Biochemical studies indicated that these health benefits are associated with the antioxidant properties of ACN compounds (Zheng et al., 2011; Wallace and Giusti, 2015). Plant-derived ACNs have been used as functional ingredients in dietary and health food supplements. The involvement of ACNs in the modulation of intestinal microbiota has been analyzed by *16S rDNA* sequence-based analyses (Chen et al., 2018; Yan et al., 2018c). The results indicated that ACNs significantly increased the relative abundances of *Bifidibacterium* and *Allisonella* (Yan et al., 2018c). A few recent studies indicated that ACNs from berries could reverse the dextran sulfate sodium-induced imbalance in gut microbiota (Chen et al., 2018; Peng et al., 2019). However, the mechanisms that underlie the impact of ACNs on the microbiota remain to be clarified, and *in vitro* culture-based analysis is required for full clarification of the interactions between ACNs and microbes.

This study performed an *in vitro*, culture-based analysis of the microbiological effects of ACN from *L. ruthenicum* Murray, a unique nutritional food widely distributed in the salinized desert of Qinghai-Tibet Plateau. As far as we are acknowledged, it is the first *in vitro* study on the interaction between probiotics and ACNs. Oxygen stress relief observed in this study might provide an explanation for ACN-induced increase of *Lactobacillus*, *Bifidobacterium* (Chen et al., 2018; Peng et al., 2019), and other anaerobes such as *Akkermansia* (Cremonini et al., 2019) in intestinal microbiota. Meanwhile, the TRX system is a key component in the antioxidant system widely distributed in lactic acid bacteria, and the enhancement of the TRX system by ACN implies its potential in growth improvement of other oxygen-sensitive probiotics.

Moreover, from the perspective of the food industry, this study provides a solution to the growth of probiotic *L. plantarum* in milk. The effect of ACN can be considered for industrial purposes and has practical implications for commercial applications of *L. plantarum* and other probiotics in the dairy industry.

## Data Availability Statement

The raw data supporting the conclusions of this article will be made available by the authors, without undue reservation, to any qualified researcher.

## Author Contributions

MY, BW, and ZL designed the experiments, analyzed the results, and wrote the manuscript. MY, BW, and XF performed the experiments with the assistance of LZ and RL. MG, GW and CY provided technique assistance and valuable suggestions for this project. All authors reviewed and approved the final version of the manuscript.

## Conflict of Interest

MY, XF, MG, CY, and ZL are employed by Bright Dairy & Food Co., Ltd. GW was employed by Bright Farming Co., Ltd.The remaining authors declare that the research was conducted in the absence of any commercial or financial relationships that could be construed as a potential conflict of interest.
